# Enhancing text pre-processing for Swahili language: Datasets for common Swahili stop-words, slangs and typos with equivalent proper words

**DOI:** 10.1016/j.dib.2020.106517

**Published:** 2020-11-10

**Authors:** Bernard Masua, Noel Masasi

**Affiliations:** College of Information and Communication Technologies (CoICT), University of Dar Es Salaam, Ali Hassan Mwinyi Road, Kijitonyama campus, Dar Es Salaam, TZ 33335, Tanzania

**Keywords:** Natural language processing, Text pre-processing, Swahili language, Stop-words, Slangs, Typos, Machine learning

## Abstract

Natural Language Processing requires data to be pre-processed to guarantee quality models in different machine learning tasks. However, Swahili language have been disadvantaged and is classified as low resource language because of inadequate data for NLP especially basic textual datasets that are useful during pre-processing stage. In this article we develop and contribute common Swahili Stop-words, common Swahili Slangs and common Swahili Typos datasets. The main source for these datasets were short Swahili messages collected from Tanzanian platform that is used by young people to convey their opinions on things that matters to them. Therefore, we derive list of common Swahili stop-words by reviewing most frequent words that are generated with Python script from our corpus, review common slang with help of Swahili experts with their corresponding proper words, and generate common Swahili typos by analysing least frequent words generated by a Python script from corpus. The datasets were exported into files for easy access and reuse. These datasets can be reused in natural language processing as resources in pre-processing phase for Swahili textual data.

## Specifications Table

**Table:** 

Subject	Computer Science, Artificial intelligence
Specific subject area	Natural Language Processing, Textual Data Pre-processing
Type of data	Table (CSV file -the list of common Swahili stop-words). Table (CSV file - the list of common Swahili Slangs). Table (CSV file - the list of common Swahili Typos).
How data were acquired	Pre-acquired Swahili text messages data from U-report SMS platform, dataset was downloaded in JSON format, we analysed texts by using Python scripts and then we reviewed filtered datasets with help Swahili expert.
Data format	Raw
Parameters for data collection	We created a dataset of common Swahili stop-words from SMS dataset by analysing most common words, their position in sentences and if removed won't affect the meaning. The Dataset of common Swahili Slangs was obtained from Swahili SMS dataset, words that are regarded as informal are listed with their respective proper Swahili word. Dataset of common Swahili Typos and their respective proper word was created by analysing Swahili SMS dataset to spot misspelt words.
Description of data collection	Common Swahili stop-words dataset consist a list of words which does not add much meaning to a sentence, hence can be ignored without sacrificing the meaning of the sentences. Lists of Swahili Slangs and typos with their respective proper words are lowercased and comma separated. Slangs/typos will be replaced by its respective proper word so as to maintain consistence during vectorization [1] to form vectors that are used in training Machine Learning algorithms [2]. Swahili data consists of SMS received from young people in Tanzania expressing their views on topic across various fields such as Health, Education, Menstrual Hygiene, Corona, WASH, Nutrition, HIV, Violence against Children, and U-Report.
Data source location	The source of the Swahili SMS data is [3]
Data accessibility	Common Swahili Stop-words Repository name: Mendeley Data Data identification number: DOI: 10.17632/mmf4hnsm2n.1 Direct URL to data: https://data.mendeley.com/datasets/mmf4hnsm2n/1 Common Swahili Slangs Repository name: Mendeley Data Data identification number: DOI: 10.17632/b8tc96xf3h.1 Direct URL to data: https://data.mendeley.com/datasets/b8tc96xf3h/1 Common Swahili Typos Repository name: Mendeley Data Data identification number: DOI: 10.17632/mmf4hnsm2n.1 Direct URL to data: https://data.mendeley.com/datasets/3xmsjhdrc9/1

## Value of the Data

• These datasets are important because they contribute to improving Swahili textual data pre-processing especially Swahili being a low resource language. For other languages such as English there are well documented resources for textual data pre-processing and can be accessed through different libraries which is not a case for Swahili.
• The datasets will benefit researchers, application developers and anyone interested in machine learning especially in natural language processing and works with Swahili textual data.
• These provided datasets can be used during data pre-processing stage for Natural Language Processing tasks such as Topic Analysis and Sentiment analysis to remove stop-words, replace slang and typos while working with any Swahili textual data.
• Also, these datasets can be updated and reused to fit into certain domain areas.

## 1 Data Description

This section provides an individual description of each dataset in the following paragraphs.

Common Swahili Stop-words; The dataset contains over 254 unique Swahili words that are regarded as Stop-words since they do not add much meaning to a sentence, hence can be ignored without sacrificing the meaning of Swahili sentences. The entire dataset is lowercased and stored in a Comma Separated Value file format with 8-bit Unicode Transformation Format. The dataset can also be saved in other formats such as Tab separated values, .TXT, Json, and others depending on how it will be used in Machine Learning tasks. We provide the dataset on the link https://data.mendeley.com/datasets/mmf4hnsm2n/1 accessible for public use.

Common Swahili Slangs; The dataset contains 2 columns and over 234 unique rows, one column for slang and other for respective Swahili proper word. All words are lowercased and stored in a Comma Separated Value file format with 8-bit Unicode Transformation Format. We provide the dataset on the link https://data.mendeley.com/datasets/b8tc96xf3h/1 publicly accessible.

Common Swahili Typos; The dataset contains 2 columns and over 431 unique rows, one column for typo and other for respective Swahili proper word. All words are lowercased and stored in a Comma Separated Value file format with 8-bit Unicode Transformation Format for easy use in machine learning pre-processing stage [4]. We provide the typo dataset updated over time on the link https://data.mendeley.com/datasets/3xmsjhdrc9/1.

Table 1 below show required steps for Python script to prepare Swahili stop-word dataset.

**Table 1:** Required steps for Python script to prepare Swahili stop-word dataset.

1. Open the corpus dataset for reading 2. Remove punctuation marks 3. Lowercased 4. Perform tokenization 5. Count word occurrence in a list of words obtained on above step 6. Generate a list of tuples for most frequent words 7. Export in text file for review

Table 2 belowine shows required steps for Python script to prepare Swahili slang dataset.

**Table 2:** Required steps for Python Script to prepare Swahili Slangs dataset.

1. Open the corpus dataset for reading 2. Remove punctuation marks 3. Lowercasing 4. Selecting random messages from each topic 5. Export each batch corresponding to each topic to its respective text file for review 6. Combining results from reviewers with already known Swahili Slangs from IKS 7. Remove duplicates based on slangs words

Table 3 below shows required steps for Python script to prepare Swahili typos dataset.

**Table 3:** Required steps for Python script to prepare Swahili typos dataset.

1. Open the corpus dataset for reading 2. Remove punctuation marks 3. Lowercased 4. Perform tokenization 5. Count word occurrence in a list of words obtained on above step 6. Generate a list of tuples for least frequent words 7. Create batches of words depending on frequencies 8. Export each batch to its respective text file for review

**Fig. 1:**
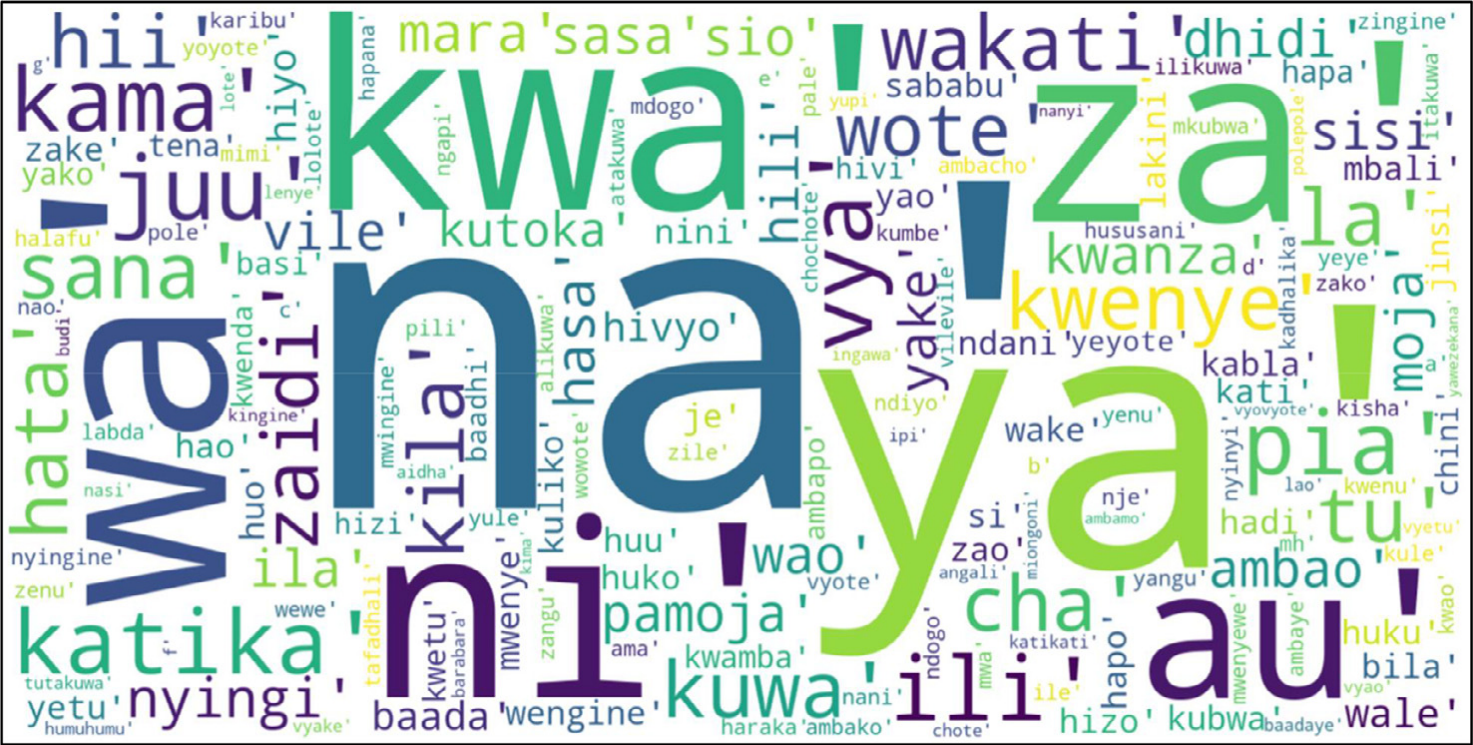
Word-Cloud for top 200 stop-words.

Fig. 1 below show a word-cloud visualization for top 200 Swahili stop-words.

**Fig. 2:**
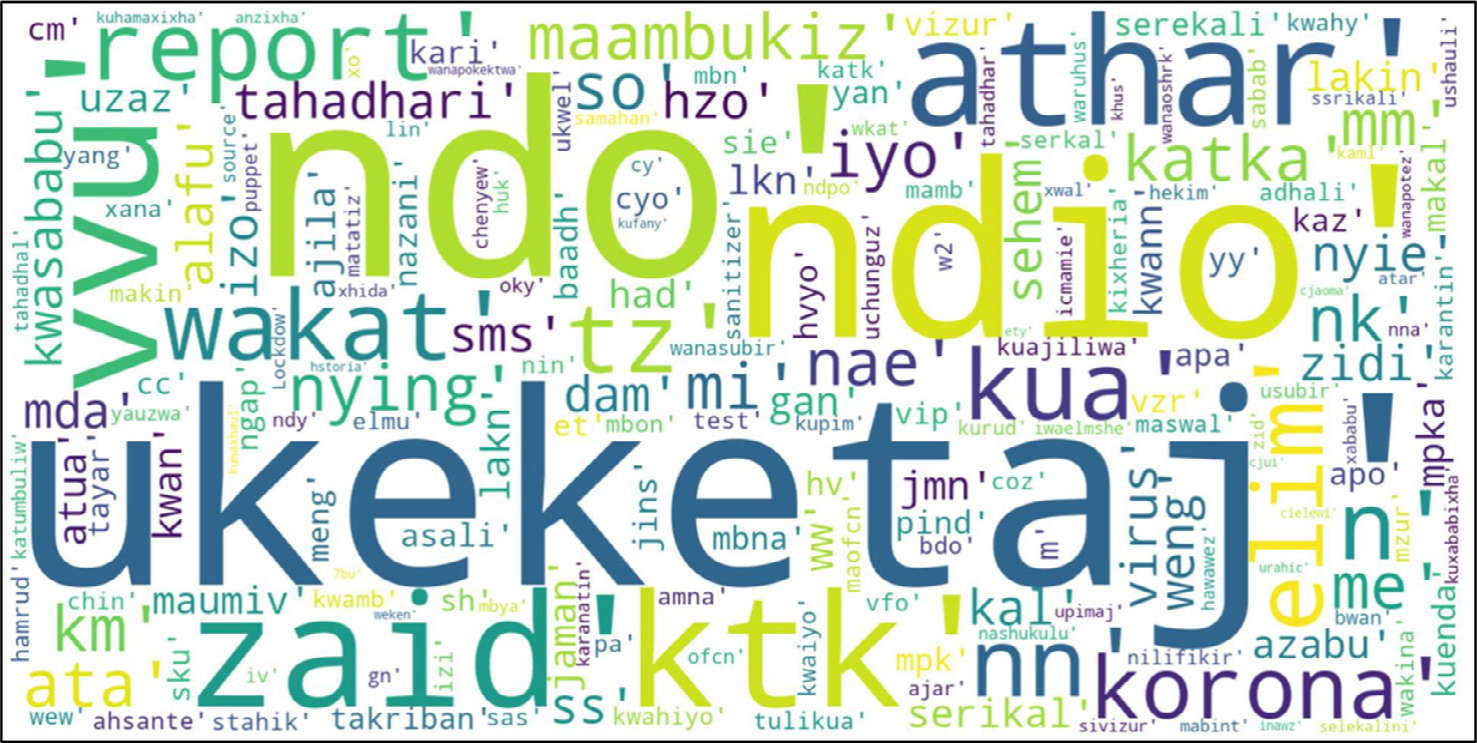
Word-Cloud for top 200 Swahili typos.

Fig. 2 below show a word-cloud visualization of top 200 Swahili typos.

## 2 Experimental Design, Materials and Methods

This section provides details on the methodology used to prepare the datasets. we describe the procedures for developing Common Swahili Stop-words dataset, Common Swahili Slangs dataset, Common Swahili Typos dataset and their respective proper words in the following subsections.

### 2.1 Preparing common Swahili stop-words dataset

We first used datasets from [3] to create a corpus which only included Swahili conversations. The collected Swahili corpus is from Tanzania SMS platform; the data was made up of 248,944 Swahili messages with a total of 4 million words and 320 thousand unique words. The corpus has a wide scope of topics that included: Health, Education, Menstrual Hygiene, Corona, WASH, Nutrition, HIV, Violence against Children, and U-Report. We obtained our dataset by processing the generated corpus as observed by [5] and [6] using a Python script which remove punctuation marks [7], lowercased [8], perform tokenization of the dataset [9], and generate a list of tuples with words and their corresponding frequencies using freqdist function from Natural Language Toolkit (NLTK) [10]. After that, we took more than 1000 most frequent words to be reviewed by Swahili experts. It was reviewed by three people including a member of Institute of Kiswahili Studies (IKS) at the University of Dar es Salaam (UDSM) to remain with only words that can be ignored without sacrificing the meaning of Swahili sentences. Required steps of our Python script is shown on Table 1. Also, we translated English stop-words [11] to Swahili, then they were reviewed with help of a member of IKS and combined with previously obtained stop-words. Finally, the resulting stop-words were exported in a text file. Fig. 1 shows a word-cloud presentation of top 200 most frequent Swahili stop-words as they appear on corpus.

### 2.2 Preparing common Swahili slangs dataset

We prepared the Swahili dataset of slangs and their respective proper Swahili words by reviewing textual data collected from a SMS platform based in Tanzania [3]. In this platform young people from all regions to express their opinion on issues they care about, connect with each other, connect with their leaders and get real-time information and feedback on new initiatives and campaigns [3]. We obtained our dataset by processing the generated corpus by using a Python script which remove punctuation marks [7], lowercase [8], then selecting 500 random messages from each topic to be reviewed with help of Swahili experts from IKS, who identifies words that are used as slangs and provide their respective proper Swahili words. The respective required steps of our Python script is shown on Table 2. The resulting dataset was then combined with already known Swahili Slangs from IKS to create this dataset.

### 2.3 Common Swahili typos dataset

We generate the common Swahili typos dataset by using datasets from [3] to create a corpus which only included Swahili conversations. The collected Swahili corpus is from Tanzania SMS platform; the data was made up of 248,944 Swahili messages with a total of 4 million words with a wide scope that included: Health, Education, Menstrual Hygiene, Corona, WASH, Nutrition, HIV, Violence against Children, and U-Report. We obtained our dataset by processing the generated corpus by using a Python script which remove punctuation marks [7], lowercased [8], perform tokenization of the dataset [9], and generate a list of tuples with words and their corresponding frequencies by using freqdist function [10]. After that, we took more than 1500 least frequent words to be reviewed with help of Swahili experts from IKS. The respective required steps of our Python script is shown on Table 3. Least frequent words were reviewed to identify common misspelled words in batches depending on their frequencies; the batches were of 5 to 10, 11 to 15 and 16 to 20 words occurrences. With help of Swahili expert from IKS we then fill in their respective proper words to generate a typos dataset. Fig. 2 shows a word-cloud visual representation of top 200 Swahili typos and the frequency in which they appear on corpus.

## Ethics Statement

The work does not involve human subject nor animals but ethical requirements for publication in Data in Brief journal are observed.

## Declaration of Competing Interest

The authors declare that they have no known competing financial interests or personal relationships which have, or could be perceived to have, influenced the work reported in this article.
